# STAT3 pathway regulates lung-derived brain metastasis initiating cell capacity through miR-21 activation

**DOI:** 10.18632/oncotarget.4742

**Published:** 2015-07-25

**Authors:** Mohini Singh, Neha Garg, Chitra Venugopal, Robin Hallett, Tomas Tokar, Nicole McFarlane, Sujeivan Mahendram, David Bakhshinyan, Branavan Manoranjan, Parvez Vora, Maleeha Qazi, Carolynn C. Arpin, Brent Page, Sina Haftchenary, David A. Rosa, Ping-Shan Lai, Rodolfo F. Gómez-Biagi, Ahmed M. Ali, Andrew Lewis, Mulu Geletu, Naresh K. Murty, John A. Hassell, Igor Jurisica, Patrick T. Gunning, Sheila K. Singh

**Affiliations:** ^1^ McMaster Stem Cell and Cancer Research Institute, McMaster University, Hamilton, Ontario, Canada; ^2^ McMaster Centre for Functional Genomics, McMaster University, Hamilton, Ontario, Canada; ^3^ Michael G. DeGroote School of Medicine, McMaster University, Hamilton, Ontario, Canada; ^4^ Departments of Biochemistry and Biomedical Sciences, McMaster University, Hamilton, Ontario, Canada; ^5^ Departments of Pathology & Molecular Medicine, McMaster University, Hamilton, Ontario, Canada; ^6^ Departments of Surgery, Faculty of Health Sciences, McMaster University, Hamilton, Ontario, Canada; ^7^ Princess Margaret Cancer Centre, University Health Network, IBM Life Sciences Discovery Centre, Toronto Medical Discovery Tower, Toronto, Ontario, Canada; ^8^ TECHNA Institute for the Advancement of Technology for Health, UHN and University of Toronto, Toronto, Ontario, Canada; ^9^ Departments of Medical Biophysics and Computer Science, University of Toronto, Toronto, Ontario, Canada; ^10^ Department of Chemistry, University of Toronto, Mississauga, ON, Canada; ^11^ Department of Medicinal Chemistry, Faculty of Pharmacy, Assiut University, Assiut, Egypt

**Keywords:** brain metastases, brain metastasis initiating cell, STAT3, miR-21

## Abstract

Brain metastases (BM) represent the most common tumor to affect the adult central nervous system. Despite the increasing incidence of BM, likely due to consistently improving treatment of primary cancers, BM remain severely understudied. In this study, we utilized patient-derived stem cell lines from lung-to-brain metastases to examine the regulatory role of STAT3 in brain metastasis initiating cells (BMICs). Annotation of our previously described BMIC regulatory genes with protein-protein interaction network mapping identified STAT3 as a novel protein interactor. STAT3 knockdown showed a reduction in BMIC self-renewal and migration, and decreased tumor size *in vivo*. Screening of BMIC lines with a library of STAT3 inhibitors identified one inhibitor to significantly reduce tumor formation. Meta-analysis identified the oncomir microRNA-21 (miR-21) as a target of STAT3 activity. Inhibition of miR-21 displayed similar reductions in BMIC self-renewal and migration as STAT3 knockdown. Knockdown of STAT3 also reduced expression of known downstream targets of miR-21. Our studies have thus identified STAT3 and miR-21 as cooperative regulators of stemness, migration and tumor initiation in lung-derived BM. Therefore, STAT3 represents a potential therapeutic target in the treatment of lung-to-brain metastases.

## INTRODUCTION

Metastases are the most common neoplasm to affect the adult central nervous system, occurring at a rate ten times greater than that of primary neural cancers [1]. Brain metastases (BM) occur late in the progression of the primary cancer, and are typically associated with poor patient prognosis and survival; even with multimodal treatment, survival is only 4–12 months [2, 3]. Lung cancer is the primary source for BM and accounts for 40–50% of cases, followed by breast cancer with 15–25% of cases, and melanoma with 5–20% of cases [4]. An increase in the incidence of BM has been recently noted, the reasons for which remain unclear but may be associated with the constantly improving treatment of the primary cancer that allows resistant cells to escape to the brain as a sanctuary site [5]. The role of a subpopulation of cells capable of tumor formation, also known as tumor initiating cells or TICs, has been extensively studied and reported in several solid primary cancers including those of the brain [6], breast [7], colon [8], and prostate [9, 10]. Our previous work [11] established the identification of TICs specifically in BM through intracranial xenotransplantation of BM from lung primary cancers. These cells exhibited properties similar to BTICs, and are indicative of a brain metastasis initiating cell (BMIC) population.

The metastatic progression of cancer involves several extremely complex but poorly understood stages. Identification and characterization of the pathways and molecules that regulate this process will thus be crucial to our understanding and subsequent treatment of BM. Several genes have been implicated in the regulation of metastases. We have identified a list of candidate genes as being significantly overexpressed in BM, as compared to primary brain and lung tumors [11]. A protein interaction network mapping of metastasis regulatory genes identified Signal Transducers and Activators of Transcription 3 (STAT3) as one of the key interactors of BM candidate genes.

The STAT family of transcription factors mediates cell communication and prompts a wide range of biological responses. Persistent activation of STAT3 has been observed in approximately 70% of cancers [12–14] and is believed to regulate TIC activity [15]. Inhibition of STAT3 has been validated *in vitro* and *in vivo* as a promising therapeutic avenue for cancer treatment, and several molecules have been identified to block STAT3 activation [16, 17]. Recent studies have implicated STAT3 as a vital regulator of microRNA (miRNA) expression, and subsequently the STAT3 signaling pathway is controlled by several specific miRNAs [18–20]. miRNAs are a class of evolutionarily conserved non-coding RNA molecules [21]. miRNAs bind to the 3′ UTR regions of target genes and suppress their expression at a post-transcriptional level, ultimately resulting in mRNA degradation or translational inhibition [22]. Iliopoulos *et al*. [18] identified transcription of miR-21 and miR-181b to be activated by STAT3, which subsequently led to the induction of a stable transformed state in cancer cell lines. Rozovski *et al*. [19] found the gene expression of several miRNAs, including miR-21, to be regulated by STAT3 in chronic lymphocytic leukemia cells. It was Loffler and colleagues [20] who discovered two phylogenetically conserved STAT3 binding sites in miR-21 that regulate its oncogenic activity.

Though lung cancer is the most common source of BM, there is very little data supporting the role of STAT3 and miR-21 in BM progression. In this study we identify STAT3 as a key regulator of lung-to-brain metastases through interaction with miR-21. We demonstrate that STAT3 knockdown can reduce self-renewal, migration, and tumor formation of a TIC population in BM. Further studies reveal that STAT3 potentially exerts its activity through miR-21, possibly through regulation of downstream tumor suppressor genes. Our studies confirm the role of STAT3 in BM development, and suggest that STAT3 may be a therapeutic target in the treatment of the lung-to-brain metastatic process.

## RESULTS

### BMIC lines exhibit stem cell properties

**Figure 1:**
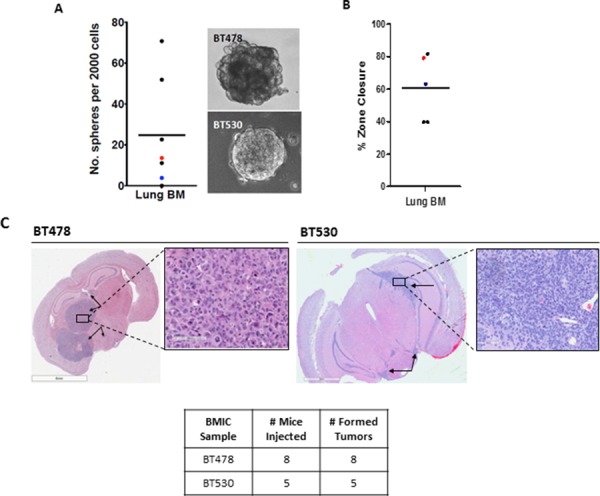
Brain metastases (BM) from the lung possess a cancer stem cell (CSC) population *in vitro* and possess tumor-initiating cell (TIC) populations *in vivo* Patient-derived BM samples were grown as tumorspheres in tumor sphere media (TSM). **A.** Self-renewal, a hallmark of CSCs, was determined though assessment of secondary sphere-forming capacity. Each dot represents a single patient sample, color dots indicate samples which have developed into sustainable patient-derived cell lines (red, BT478; blue, BT530); bar indicates mean; insets are representative bright field images of spheres of BT478 and BT530. **B.** Migration, a key trait of metastatic cells, was determined through zone exclusion assays. Each dot represents a single patient sample, coloured dots indicate samples which have developed into sustainable patient-derived cell lines (red, BT478; blue, BT530) bar indicates mean). **C.** NOD-SCID mice were used in all experiments; injected cells were cultured as tumorspheres. TIC capacity is demonstrated through tumor growth in the brain, when injected intracranially *p < 0.05 (100,000 cells, *n* = 5 for BT478, *n* = 4 for BT530).

Brain metastasis initiating cells (BMICs) isolated from human primary lung-derived brain metastasis samples BT478 and BT530 were propagated as tumorspheres in in tumor sphere medium (TSM). Both the cell lines were shown to possess self-renewal capacity and migratory potential as assessed by secondary sphere formation (Figure 1A) and zone exclusion assays (Figure 1B), respectively. Both BMIC lines showed differential expression of surface markers such as CD133 and EpCAM, as analyzed by flow cytometry (Supplementary Figure 1A). CD133 has been used to prospectively identify brain tumor initiating cells (BTICs) [6] as well as cancer stem cell populations in other primary tumors [23, 24]. EpCAM is an epithelial cell marker overexpressed in carcinomas of various origins [25]. The tumor-initiating capacity of both BMIC lines used in this study was assessed through intracranial injections into NOD-SCID mice (Figure 1C), where both BMIC lines were capable of tumor formation with as few as 100 cells injected, though the tumour size and aggressiveness differed between both samples. Together these data confirm the presence of a TIC population in both BMIC lines tested.

### STAT3 is a putative BMIC regulatory gene

**Figure 2:**
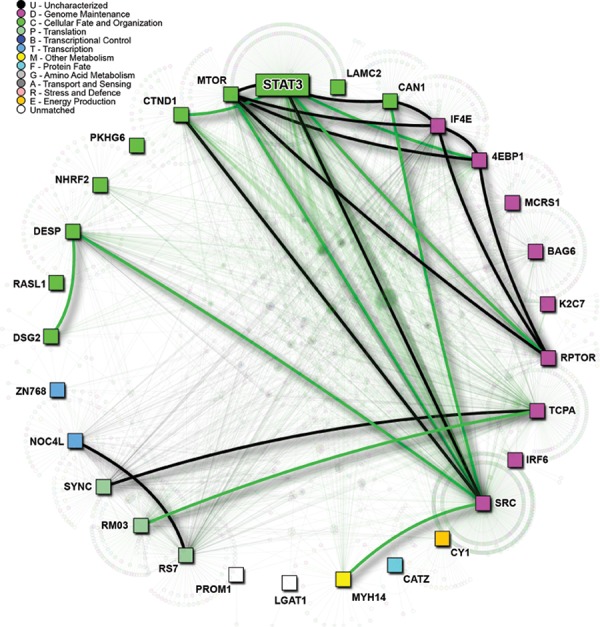
Protein connectivity mapping implicates STAT3 as a putative BMIC regulatory gene Protein-protein interaction network of putative BMIC regulatory genes. Black lines represent known interactions; green lines signify predicted, and thus novel interactions. Direct interactions among BMIC genes is highlighted by thicker edges. Gene Ontology (GO) biological function is represented by node color, as per legend.

Previous work in our lab utilized RNA-sequencing to compare gene expression of lung-to-brain metastases to primary brain tumor and to primary lung tumor samples, and led to the identification of 30 genes upregulated specifically in the lung derived brain metastases [11]. These genes, termed BMIC regulatory genes, were annotated with known and predicted physical protein interactions using I2D V2.3 [26] and FpClass V1.0 [27]. We found that Activators of Transcription 3 (STAT3) was a novel and direct interactor in the BMIC regulatory network (Figure 2). STAT3 has already been shown to be persistently activated in a variety of cancers, and is believed to regulate multiple cancer stem cell populations including those that may drive primary brain tumors such as glioblastoma. STAT3 is required for proliferation and maintenance of multi-potency in glioblastoma stem cells [15].

### STAT3 functions to regulate self-renewal and tumorigenicity of BMICs

**Figure 3:**
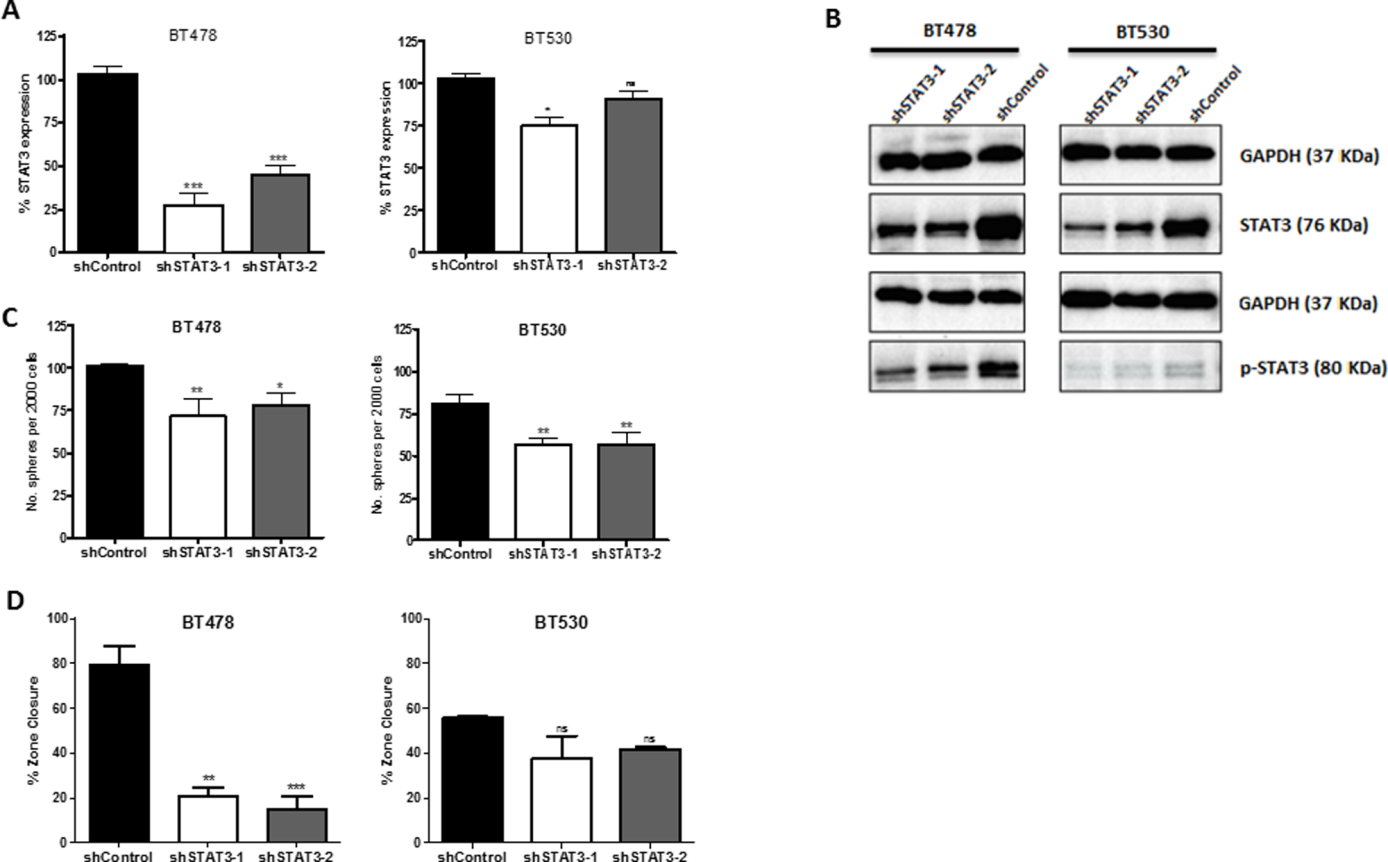
Knockdown of STAT3 demonstrates potential regulatory role in self-renewal and metastasis Tumorspheres were transduced with short-hairpin lentiviral vectors against candidate BMIC regulatory gene STAT3. **A.** STAT3 transcript levels by qRT-PCR reveal significant knockdown in brain metastases achieved by two different shSTAT3 vectors as compared to the shControl. **B.** Protein levels of STAT3 and phosphorylated STAT3 in control and knockdown samples by Western blot, relative to a GAPDH control. **C.** Self-renewal was assessed through sphere formation per 2000 cells; knockdown of STAT3 corresponded with decreased sphere formation. **D.** Zone-exclusion assays showed decreased migratory capability with STAT3 knockdown. ns non-significant; *p < 0.05; **p < 0.01; ***p < 0.001 (1-way ANOVA).

**Figure 4:**
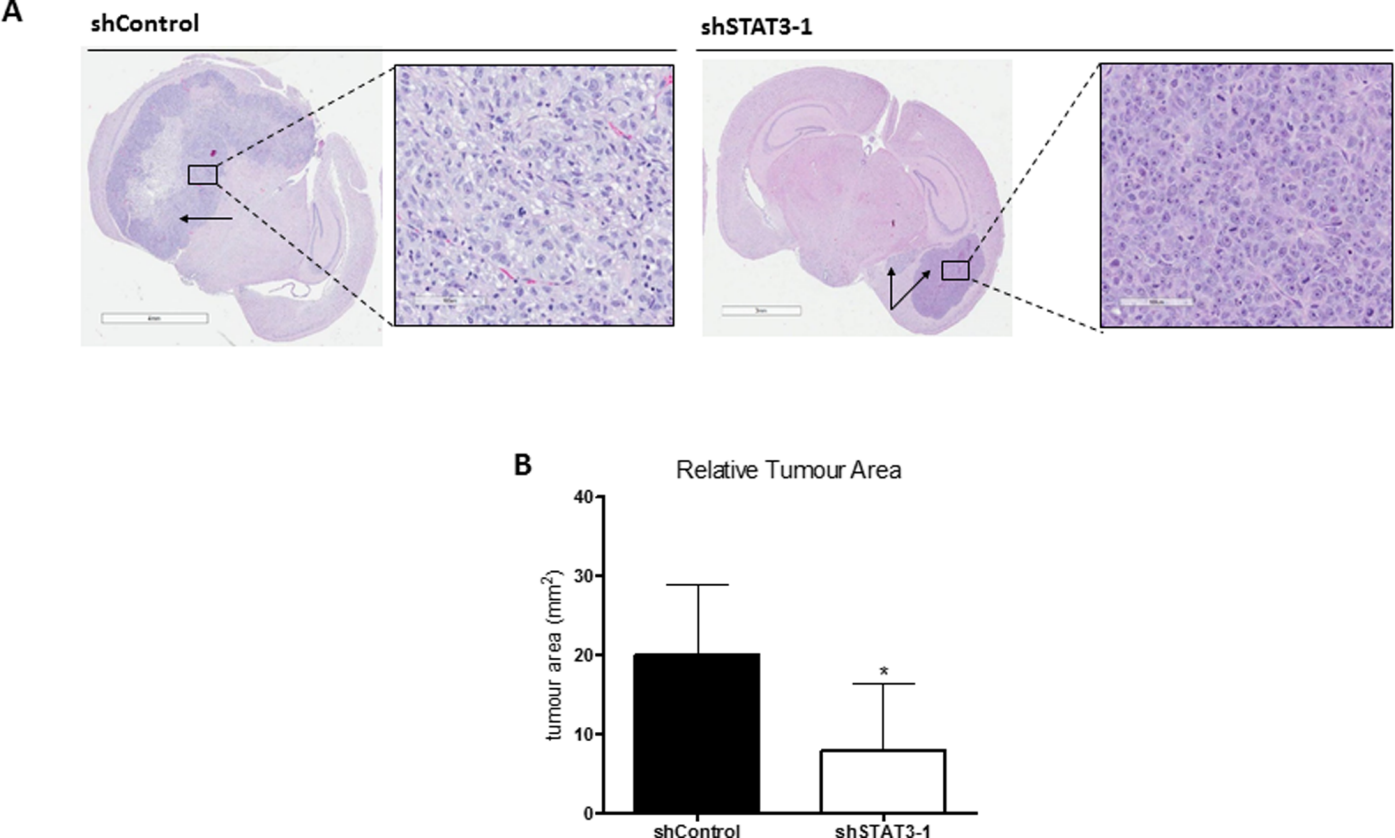
Knockdown of STAT3 demonstrates potential regulatory role in self-renewal and tumor formation **A.** 100,000 cells of shSTAT3–1 or shControl were injected into the frontal lobes of NOD-SCID mice (*n* = 3 in each group). Mice were sacrificed upon reaching endpoint. H&E sections of the brains are shown. shSTAT3 cells formed smaller tumors than shControls (arrows indicate tumors). **B.** shSTAT3–1 cells formed tumors approximately 60% smaller as compared to shControl mice. *p < 0.05 (*t* test).

To interrogate the functional significance of STAT3 in lung-derived brain metastasis, we performed lentiviral-mediated shRNA vector knockdown (KD) of STAT3 in BMIC lines. Scrambled shRNA (shControl) served as a control. The efficiency of STAT3 KD was validated at transcript (Figure 3A) and protein levels including the active phosphoform (Figure 3B) by RT-PCR and Western blotting respectively. shSTAT3–1 showed the most efficient KD and was chosen for further study. Knockdown of STAT3 corresponded with a reduction of BMIC self-renewal and migration, as seen with a decrease in sphere formation capacity (Figure 3C) and zone closure (Figure 3D). Furthermore, we also implemented *in vivo* studies in order to investigate the tumorigenic potential of STAT3 KD BMICs. We performed intracranial injections of BT478 into NODSCID mice brains and found that STAT3 KD formed tumors approximately 60% smaller than control tumors, which generated much larger and infiltrative tumors (Figure 4). Our data thus implicates STAT3 as an important regulator of self-renewal, migration and tumorigenicity in BMIC populations.

### STAT3 inhibitors impede tumor formation in NOD-SCID xenograft model

**Figure 5:**
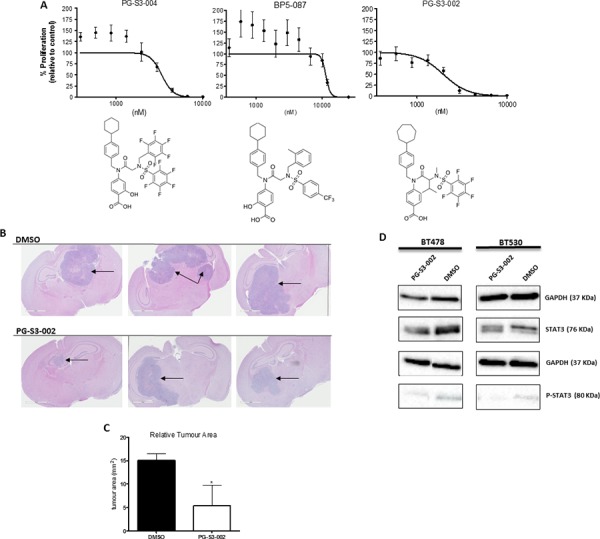
STAT3 inhibitors as candidate drugs for targeting tumor formation in brain metastases **A.** BT478 tumorspheres were treated with several STAT3 inhibitors and IC_50_ curves generated from inhibition of cell proliferation (PrestoBlue assay). **B.** BT478 tumorspheres were treated *ex vivo* with 6 μM (IC_90_) PG-S3–002 or DMSO control for 4 days. 100, 000 cells were injected into the frontal lobes of NOD-SCID mice (*n* = 3 in each group). After 4 weeks, mice were sacrificed. H&E sections of the brains are shown. **C.** PG-S3-002 treated cells formed tumors approximately 60% smaller than controls. **D.** Western blot of STAT3 and p-STAT3 protein levels after treatment of BT478 and BT530 with PG-S3–002 at IC_90_ and IC_50_ (respectively) or DMSO for 4 days. *p < 0.05 (*t* test).

BMIC line BT478 showed varied sensitivity to the STAT3 inhibitor library (Figure 5A), amongst which PG-S3–002 showed enhanced potency. To assess the clinical utility of STAT3 inhibitor PG-S3–002, BT478 was treated with PG-S3–002 at IC_90_ or DMSO after which 1 × 10^5^ viable cells, representing treatment-refractory BMICs, were injected intracranially into NOD-SCID mice. After 4 weeks, mice were sacrificed. PG-S3–002- treated cells reduced tumor formation by approximately 60% as compared to control tumors, which is similar to tumors formed by STAT3 KD (Figure 5B). The efficiency of PG-S3–002 in blocking STAT3 activity was validated by Western blot, where treatment of BT478 and BT530 with PG-S3–002 at IC_90_ and IC_50_ (respectively) reduced both STAT3 and the active phosphoform as compared to the DMSO treated control (Figure 5C).

### miR-21 as the target of STAT3

**Figure 6:**
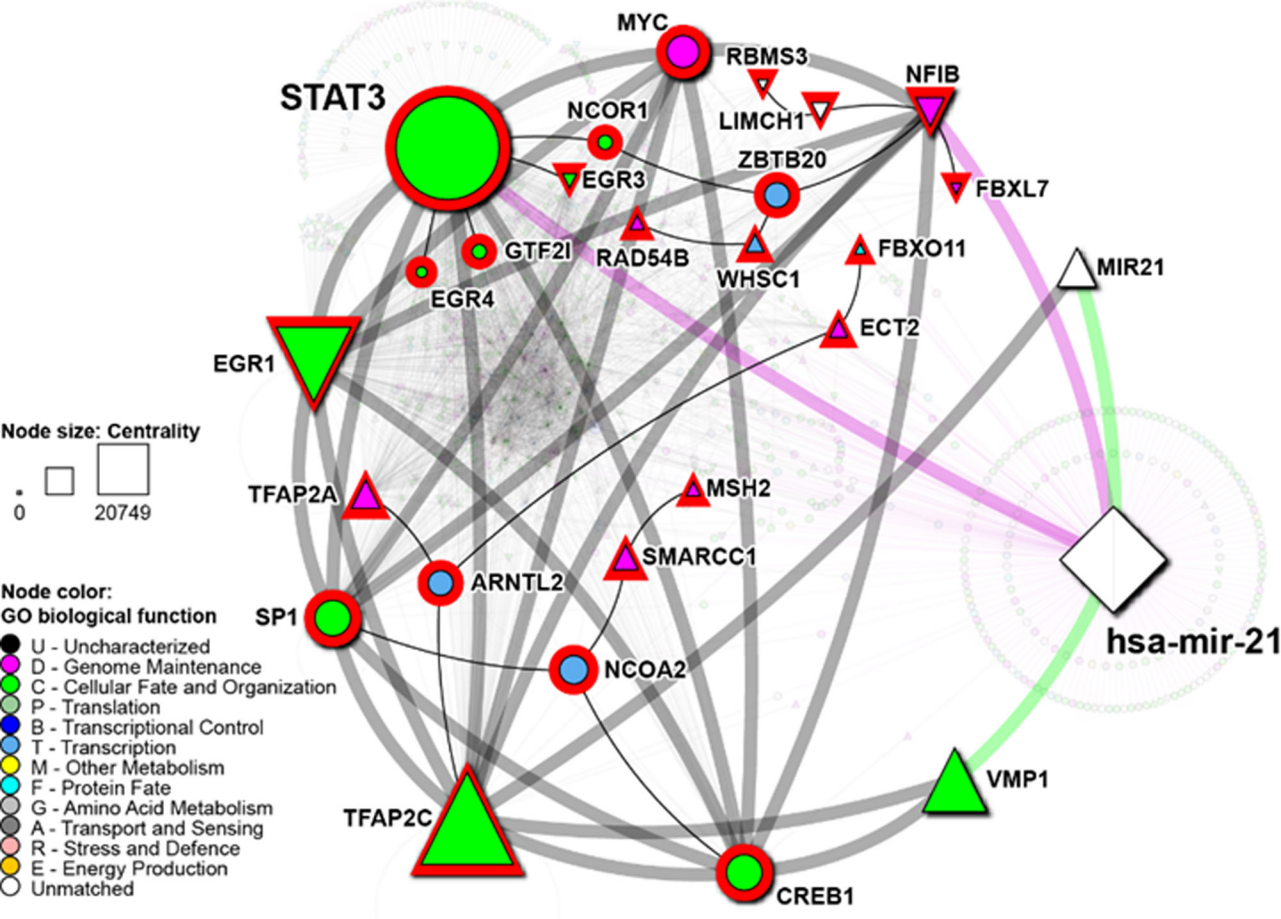
Network of STAT3 and hsa-mir-21 regulatory targets STAT3, hsa-mir-21 and their regulatory targets are represented by nodes, while regulatory relationships among these are represented by edges. hsa-mir-21 is represented by white diamond. Genes are color according to their biological function. Green edges link hsa-mir-21 to its precursor MIR21 and related gene *VMP1*. Grey edges represent TF: target relationships, magenta edges represent hsa-mir-21:target relationships. Shape of the nodes denotes expression status of genes from the meta-analysis of gene expression profiles in NSCLC. Downward oriented triangles denote downregulation, while those pointing up denote upregulation. Circles denote no significant differential expression. Size of the nodes corresponds to its centrality, measured by network betweenness. Red node highlight signifies putative TFs.

As previously described, miR 21 promoter has two putative STAT3 binding sites [20]. Additionally, it has also been demonstrated that STAT3 directly binds to the miR21 promoter and modulates its expression [18]. Hence we wanted to explore the STAT3 and hsa-mir-21 regulatory network and identify its potential targets by collating data from four different TF databases and miRDip as described in the methods. We found that both molecules are strongly interrelated (Figure 6). The regulatory potential of both molecules exists through transcriptional regulatory relationships between their targets.

### Inhibition of miR -21 reduces BMIC self-renewal and proliferation

**Figure 7:**
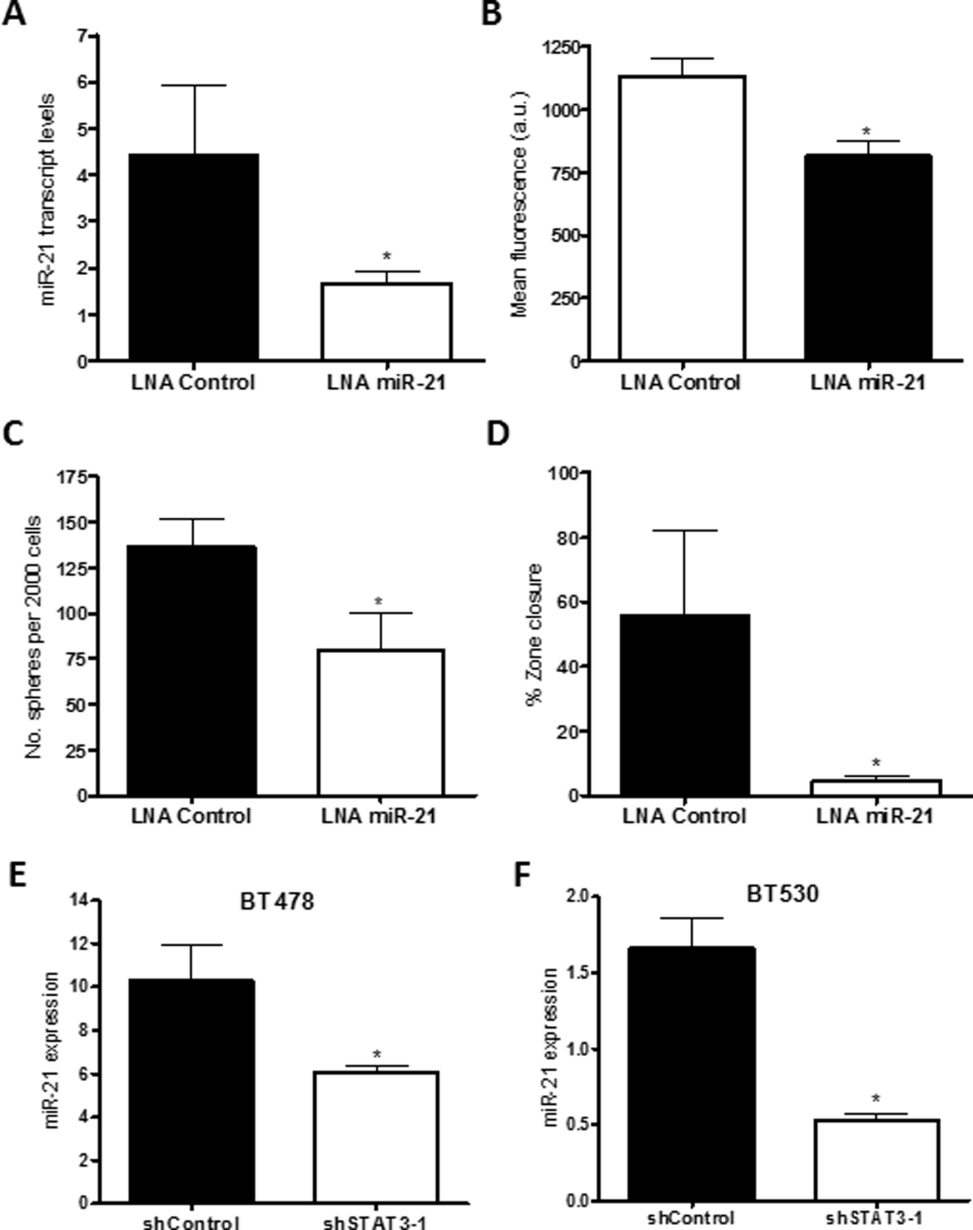
Knockdown of miR-21 produces similar reduction in self-renewal and migration *in vitro* miR-21 was knocked down using a locked nucleic acid (LNA) oligonucleotide in the BMIC line BT478. **A.** miR-21 transcript levels by qRT-PCR were moderately reduced, relative to the U6snRNA control. **B.** Self-renewal was assessed through sphere formation per 2000 cells; miR-21 knockdown corresponded with decreased sphere formation. **C.** miR-21 knockdown resulted in decreased proliferation (PrestoBlue assay). **D.** Zone-exclusion assays showed decreased migratory capability with inhibited miR-21. miR-21 transcript levels in STAT3 knockdown cells for both BMIC lines **E.** BT478 and **F.** BT530 were assessed by qRT-PCR, where miR-21 transcript levels relative to the U6snRNA control were significantly lower in shSTAT3 cells as compared to the shControl. *p < 0.05.

To evaluate the functional significance of miR-21 in BMIC populations, cells were transfected with a miR-21 inhibitor (LNA miR-21) and scrambled LNA control. Knockdown of miR-21 as confirmed by RT-PCR (Figure 7A) resulted in reduced BMIC proliferation (Figure 7B), self-renewal (Figure 7C) and cell migration (Figure 7D).

### Overexpression of miR -21 increases BMIC self-renewal and proliferation

**Figure 8:**
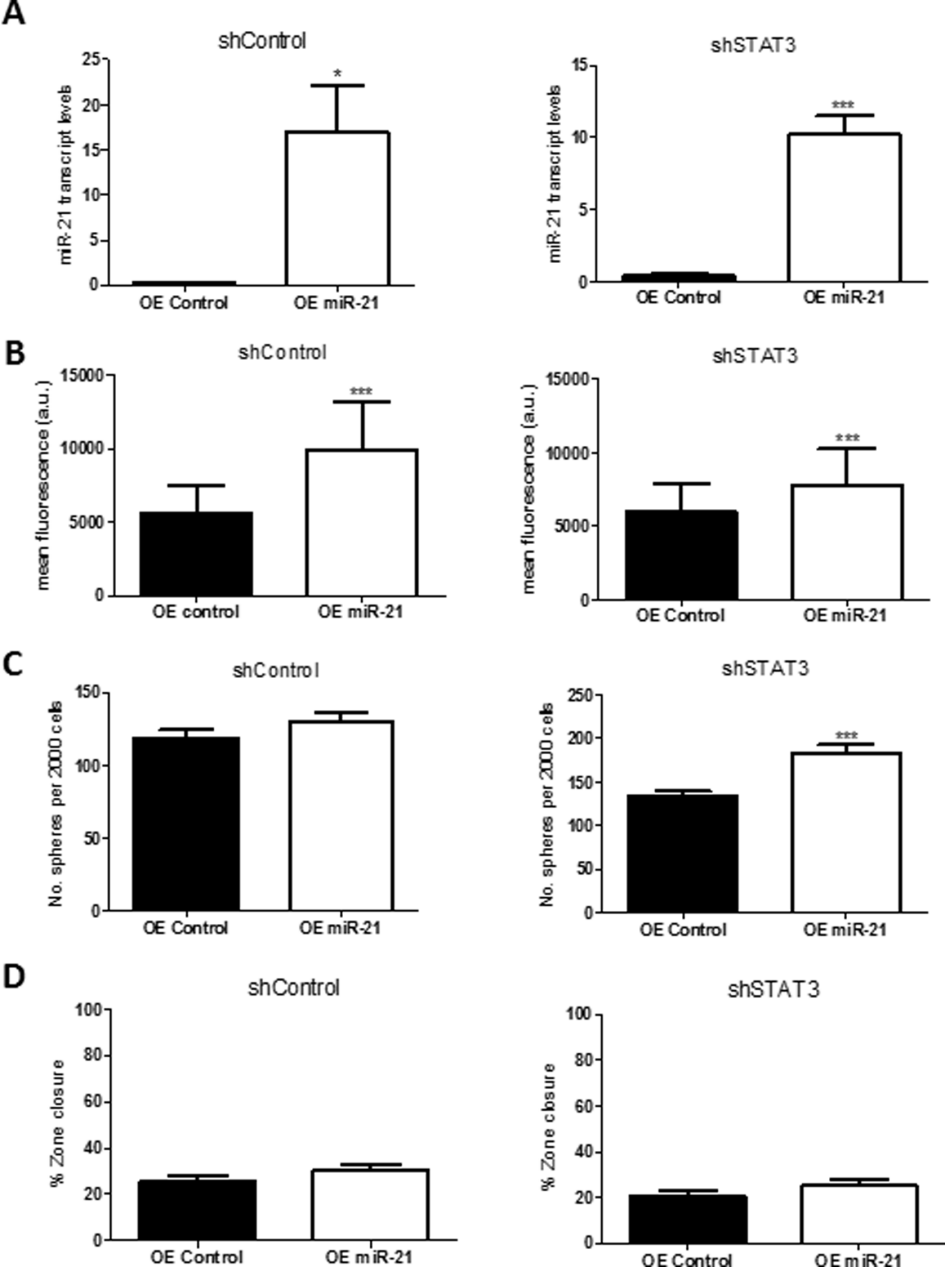
Overexpression of miR-21 rescues STAT3 knockdown, showing increased self-renewal and migration *in vitro* miR-21 was overexpressed in BT478 shSTAT3 or shControl cells with a miRIDIAN microRNA human has-miR-21–5p mimic or a negative control. **A.** miR-21 transcript levels by qRT-PCR were greatly increased in the miR-21 overexpressed cells as compared to the negative control, relative to the U6snRNA control. **B.** miR-21 overexpression resulted in increased proliferation (PrestoBlue assay) as compared to the negative control. **C.** Self-renewal was assessed through sphere formation per 2000 cells; miR-21 overexpression corresponded with increased sphere formation as compared to the negative control. **D.** Zone-exclusion assays showed mild migratory capability with overexpression of miR-21 as compared to the control. *p < 0.05; **p < 0.01; ***p < 0.001

To confirm the role of miR-21 in regulating STAT3 activity, BT478 BMICs with STAT3 knockdown were transfected with a miR-21 mimic (OE miR-21) and mimic control. Overexpression of miR-21 as confirmed by RT-PCR (Figure 8A) resulted in increased BMIC proliferation (Figure 8B), self-renewal (Figure 8C) and slight increase in cell migration (Figure 8D).

### miR-21 is overexpressed in lung cancer patients and predicts poor survival

**Figure 9:**
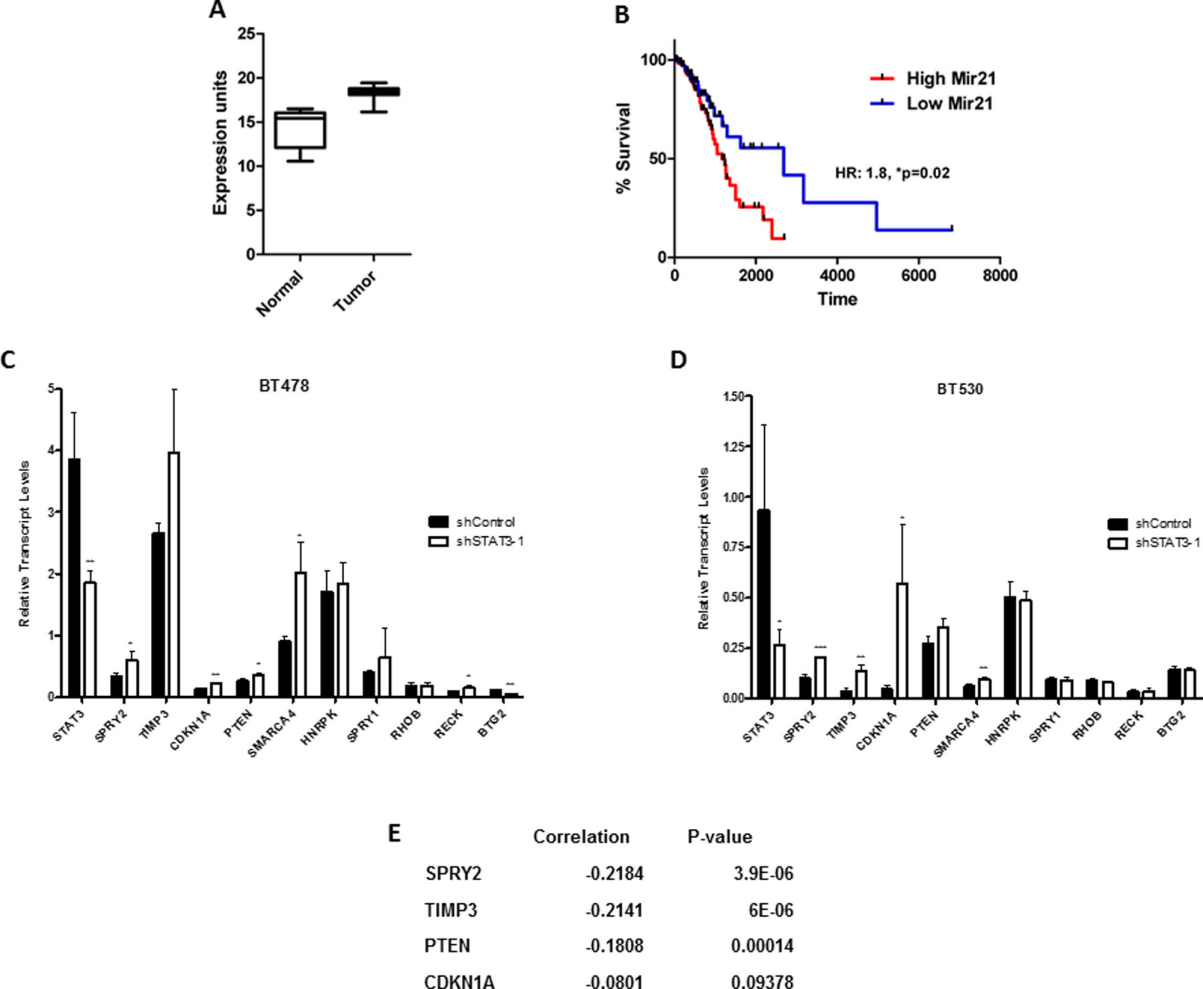
miR-21 expression in lung adenocarcinoma patients and its association with STAT3 **A.** Examination of gene expression profiling obtained from TCGA identified a significant upregulation of miR-21 in lung adenocarcinoma samples as compared to normal lung. (*p* < 0.0001). **B.** Patients with high miR-21 expressing tumors had poorer overall survival. (*p* = 0.02). qRT-PCR analysis of target genes downstream of miR-21 in STAT3 KD in BMIC lines **C.** BT478 and **D.** BT530. **E.** The calculated Pearson distance between miR-21 and genes identified those that were inversely co-expressed with miRNA-21 by gene expression analysis in lung adenocarcinoma samples (TCGA). These genes showed significant upregulation in the STAT3 KD as compared to the control. ns non significant; *p < 0.05; **p < 0.01; ***p < 0.001 (multiple *T* tests).

Our observations suggesting that miR-21 regulated key biological characteristics of aggressive lung cancer samples prompted us to examine the expression of miR-21 in a large cohort of lung cancer patients. Briefly, we obtained gene expression profiling from 420 lung adenocarcinoma, and 18 normal lung samples, from the cancer genome atlas (TCGA) lung project. Relative to normal lung, miR-21 was dramatically upregulated in lung adenocarcinoma (Figure 9A, *p < 0.0001). Given that 214 of the tumor samples had clinically annotated outcome data, we also examined whether miR-21 expression was associated with patient survival. Using the mean expression level of miR-21 to stratify patients into miR-21 high and low expression groups, we observed that patients whose tumors had high miR-21 expression experienced substantially poorer overall survival than those whose tumors expressed low levels of miR-21 (Figure 9B, HR: 1.8, *p = 0.02). Although the 5-year survival of the low expression group was 55%, the 5-year survival of the mir-21 high expression group was a dismal 25% (Supplementary Table 5). Additionally, we also validated the upregulation of mir-21 in lung cancer with 12 other miRNA profiling studies and found that it is significantly upregulated compared to normal tissue. Overall, these data support our observations that miR-21 expression is associated with hyper-aggressive lung tumors, likely due to enhanced metastatic propensity.

### STAT3 exerts its activity *via* miR-21 in BMIC cells

We observed that miR-21 transcript levels were downregulated in BMICs when STAT3 was knocked down (Figure 7E and 7F). Using results of meta-analysis of gene expression profiles in NSCLC, we examined the expression status of STAT3/hsa-mir-21 targets. We found that the majority (232 out of 451) of transcriptional targets of STAT3 are consistently downregulated, despite expression of STAT3 itself remaining stable. At the same time, only 37 out of 289 targets of hsa-mir-21 are downregulated, despite the fact that hsa-mir-21 itself is consistently reported as highly upregulated in NSCLC (Supplementary Table 5). This allows us to hypothesize that elevated expression of hsa-mir-21, rather than causing detectable expression changes of its direct targets, decreases expression of certain transcription factors by disrupting their translation.

Therefore, to better understand the role of the STAT3-miR-21 network, we tested the potential downstream targets of miR-21 using a list of published genes in the literature (Supplementary Table 6) for various malignancies. We evaluated their transcript expression levels in shSTAT3 or shControl BMIC lines (Figure 9C and 9D). Genes significantly upregulated upon STAT3 knockdown were *SPRY2*, *TIMP3*, *PTEN* and *CDKN1A*. Intriguingly, we also found an inverse correlation of miR-21 with *SPRY2* and *TIMP3* (Figure 9E) in gene expression profiles of lung adenocarcinoma samples from TCGA, emphasizing the importance of studying STAT3-miR-21 interactions in BM.

## DISCUSSION

**Figure 10:**
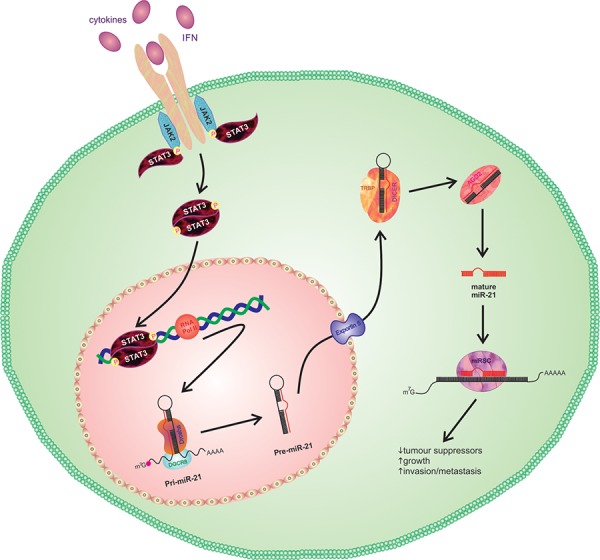
Representative schematic of STAT3 activation of miR-21 Binding of appropriate cytokines or interferons to the cell surface receptor triggers activation of Janus Kinases (JAKs), which in turn phosphorylates the tyrosine residue of bound STAT3 proteins. The phosphotyrosines mediate the dimerization of STAT3, where activated dimers will translocate to the nucleus to activate transcription of pri-miR-21. Cleavage of pri-miR-21 by Drosha will generate a precursor miR-21, or pre-miR-21, which is translocated to the cytoplasm and further processed by a Dicer complex to produce mature miR-21. miR-21 will be integrated into the RNA-induced silencing complex (RISC) and interact with target mRNA to reduce expression of tumor suppression genes such as *PTEN* and *CYLD*.

Metastases are the cause of 90% of all deaths from cancer [28], with metastasis to the brain occurring in approximately 20–40% of patients with systemic cancer [29, 30]. Despite the known complexity of the metastatic process, there remains a lack of knowledge concerning the molecular mechanisms that govern BM formation. Previous work in our lab has identified a TIC population in lung-to-brain metastases [11] and suggests the presence of a subgroup of cells capable of BM formation, termed BMICs. In this study we show that the STAT3 pathway is upregulated in these BMICs. To the best of our knowledge this is the first reported study to implicate STAT3 in lung-to-brain metastasis using patient-derived BMICs and both *in vitro* and *in vivo* experimental approaches, and we suggest a regulatory pathway involving STAT3 control of miR-21 (Figure 10).

STAT3 is involved in a wide range of physiological processes, regulating transcriptional activity in inflammation, cell survival, and stem cell maintenance [12, 31]. EGFR and KRAS mutations, activation of Src kinase, and elevated expression of interleukin-6 (IL-6) are among several molecular aberrations have been identified in lung cancer that often result in the activation of STAT3 [32–34]. Downstream targets of STAT3 enhance invasive cell properties and promote metastasis in lung cancer [35–37]. Constitutive activation of STAT3 in both lung cancer cell lines [33] and lung cancer tissues [38] has been implicated in the malignant progression of lung cancer [39] and high expression of STAT3 and phosphorylated-STAT3 are strong predictors of poor patient prognosis [40, 41].

The oncogenic potential of miR-21 has been extensively studied in a variety of hematological malignancies and primary solid tumors [42] and has been shown to regulate metastasis in breast cancer [43, 44], prostate cancer [44], colorectal cancer [45] and melanoma [46]. miR-21 was found to be overexpressed in lung cancer [47–50], and predicts poor patient survival [48, 49]. In recent studies down-regulation of miR-21 inhibited tumor cell proliferation, migration, and invasion and induced apoptosis *in vitro* [47, 50, 51] and suppressed tumor growth *in vivo* [47]. STAT3 upregulation of miR21 has been documented in breast cancer [18], nasopharyngeal carcinoma [52], and hepatocellular cancer [53], and now shown in the present study relating to lung derived BMs.

STAT3 is recognized as a central regulator in the metastatic process [16, 35], with several STAT3 inhibitors reported [54–57], yet to date none have reached the clinic. Gunning *et al*. has developed libraries of STAT3 inhibitors [17, 58, 59]; one such compound, BP-1–102, was found to inhibit growth of human breast and lung tumor xenografts [60]. The compounds screened in this study are analogues of BP-1–102, and displayed moderate IC_50_ values and efficacy *in vitro*. Though treatment of BMICs with PG-S3–002 resulted in reduced STAT3 activity *in vitro* and reduced intracranial tumor growth *in vivo*, implicating STAT3 as a druggable target in BM development, further studies will need to be conducted with *in vivo* metastasis models to confirm the ability of STAT3 inhibitors in blocking the metastatic process.

In our study knock down of miR-21 lead to decreased migration potential of BMICs (Figure 7) while overexpression of miR-21 in BMICs with shSTAT3 rescued the effects seen with STAT3 knockdown (Figure 8), suggesting that miR21 promotes brain metastatic potential. Additionally, we propose that miR-21 protects BMICs from apoptosis, regulates invasion by controlling matrix metalloproteinase inhibition and promotes cell proliferation by regulating genes such as *SPRY2*, *TIMP3*, *CDKN1A*, *SERPINB5* and *PTEN*. These, together with other analyzed genes, were previously identified as targets of miR-21 in a variety of cancers [43–45, 61–63]. Although *HNRPK*, *SPRY1*, *RHOB*, *RECK* and *BTG2* are reported to be regulated by miR-21, we did not observe any significant effect on these genes in BMICs. These findings are suggestive of many additional molecular mechanisms downstream of miR-21 and STAT3 that operate at different stages of the metastatic process, in keeping with the nature of miRNAs to target over 100 genes in different cellular systems [64]. These mechanisms support miR-21 targeting as a potentially effective strategy to block tumor metastasis.

As shown in Figure 6, STAT3 was found to be a direct target of hsa-mir-21. Concurrently, the hsa-mir-21 precursor gene is targeted by TFAP2C, which is itself a target of transcriptional control of STAT3. TFAP2C also targets the closely related gene *VMP1*, downstream of which this precursor gene is located [65]. Although targets of STAT3-mediated transcriptional control overlap only slightly with those of hsa-mir-21, STAT3 and hsa-mir-21 form an interesting negative feedback loop.

## CONCLUSIONS

Our study suggests that coordination between STAT3 and miR-21 regulate the metastatic behavior of BMICs by promoting migration and self-renewal of tumor stem cell populations, tumor cell proliferation, survival and migration. Blocking the STAT3-miR-21 pathway could form a strong rationale for a novel therapeutic approach in patients with lung-to-brain metastasis.

## MATERIALS AND METHODS

### Primary tumorsphere culture

Lung derived brain metastasis samples were obtained from consenting patients, as approved by the Research Ethics Board at Hamilton Health Sciences. Tumors were processed and maintained in TSM as described previously [66]. Of the two BMIC lines developed only BT478 was used to continue in depth *in vivo* experiments due to its high rate of engraftment.

### Flow cytometric analysis

BMICs underwent flow cytometric analysis as previously described [66]. BMICs were labelled with anti-CD133, EpCAM, or a matched isotype control (see Supplementary Table 1).

### Protein-protein interaction network

BMIC regulatory genes were mapped to proteins and their direct physical interactions were identified using I2D V2.3 [26, 67] and FpClass V1.0 (http://ophid.utoronto.ca/fpclass; [68]. Protein-protein interaction network was visualized using NAViGaTOR version 2.3.1 [69, 70].

### Lentivirus preparation and transduction

Lentiviral vectors expressing shRNA that target human STAT3 (target sequence: shSTAT3–1 5′-TGCATGTCTCCTTGACTCT-3′, shSTAT3–2 5′-TACCTAAGGCCATGAACTT-3′) and control scrambled shRNA vector were purchased from Thermo Scientific. Replication-incompetent lentivirus was produced as previously described [71] BMIC lines were transduced with lentiviral vectors and treated with puromycin after 48 hours of transduction to develop stable shSTAT-3 lines.

### Overexpression and knockdown of miR-21

miR-21 overexpression was carried out by transfecting BT478 shSTAT3 or shControl cells with a synthetic miRIDIAN mimic (has-miR-21–5p, code: C-300492-03-0005) or a negative control (miRIDIAN mimic negative control, code: CN-001000-01-05) (Dharmocon) at a final concentration of 100 nM using Lipofectamine 2000 for 24 hours. After 4 days secondary sphere formation, proliferation, and zone exclusion migration assays were plated. Antagomir-mediated miRNA knockdown was carried out by transfecting BT478 with a mirVana miRNA inhibitor (Hsa-miR-21-5p, Cat # 4464084) or negative control (scrambled miRVana miRNA inhibitor negative control #1, Cat# 4464076) (Ambion, Life technologies) at a final concentration of 50 nM for 48 hours. miR-21 expression levels after knockdown and overexpression were determined by qRT-PCR.

### Reverse transcription and quantitative PCR of mRNA and mature miRNA

For both mRNA and miRNA quantification, total RNA was isolated using Norgen RNA extraction kit (Biotek). For mRNA analysis, total RNA was reverse transcribed using qScript cDNA Super Mix (Quanta Biosciences) and a C1000 Thermo Cycler (Bio-Rad). qRT-PCR was performed using the Cfx96 (Bio-Rad) with SsoAdvanced SYBR Green (Bio-Rad) using gene specific primers (Supplementary Table 2) and GAPDH as the internal control. For miRNA analysis, total RNA was reverse transcribed using Taqman MicroRNA Reverse Transcription kit (Applied Biosytems) as described previously [72]. qRT–PCR analysis was performed using TaqMan probes (miR-21-5p, Cat#4427975; U6snRNA, Cat#4427975) and Taqman Universal Master Mix II (Cat#4440040) according to manufacturer’s instructions (Life Technologies). miRNA quantification was expressed, in arbitrary units, as the ratio of the sample quantity to the calibrator.

### Western immunoblotting

Denatured protein (30 μg) was separated as previously described [73]. Primary antibodies used were as follows: anti-STAT3 (1:1000; mouse IgG2a; Cell Signaling#9139), anti-phosphorylated STAT3 (mouse IgG1; 1:1000; Cell Signaling#4113), anti-GAPDH (mouse; 1:40, 000; Abcam#ab8245). The secondary antibodies were horseradish peroxidase-conjugated goat anti-mouse IgG (Bio-Rad) or goat anti-rabbit IgG (Sigma). Bands were visualized using Chemidoc.

### Secondary sphere formation assay

After primary sphere formation was noted, spheres were dissociated to single cells and replated in TSM as previously described [74]. Stem cell frequency was quantified by calculating the rate of secondary sphere formation from 2000 dissociated single cells.

### Zone exclusion migration assay

BMIC spheres were dissociated to single cells and replated at a density of 30,000 cells per well in a 96 well plate TSM containing 1% FBS and a 1% agar drop in the center of the well. After 24 hours to allow cell adherence, the agar drop was removed, the wells washed gently with PBS to remove floating cells, and media replaced with TSM. Migration into the empty zone was monitored over 3 days, with time points taken at day 0 and day 3.

### Cell proliferation assay

Single cells were plated in a 96-well plate at a density of 1,000 cells/200 μL per well in quadruplicate and incubated for five days. 20 μL of Presto Blue (Invitrogen), a fluorescent cell metabolism indicator, was added to each well approximately 2 h prior to the readout time point. Fluorescence was measured using a FLUOstar Omega Fluorescence 556 Microplate reader (BMG LABTECH) at excitation and emission wavelengths of 535 nm and 600 nm respectively. Readings were analyzed using Omega analysis software.

### *In vivo* BMIC intracranial injections and H&E staining of xenograft tumors

All experimental procedures involving animals were reviewed and approved by the Animal Research Ethics Board (AREB). Intracranial injections were performed as previously described [6]. Briefly, 10 μL of cell suspension was injected into the right frontal lobe of 8–10 week old NOD/SCID mice (*n* = 3). Mice were monitored weekly for signs of illness, and upon reaching endpoint, brains were harvested and embedded in paraffin for hematoxylin and eosin (H&E) staining. Images were scanned using an Aperio Slide Scanner and analyzed by ImageScope v11.1.2.760 software (Aperio).

### Screening and *ex vivo* treatment of bmics with stat3 inhibitors

A library of known direct-binding STAT3 inhibitors (BP-1–102 [75, 76], BP-5–087 [75, 76], PG-S3–002, and PG-S3–004; Supplementary Figure 1) was provided by Dr. Patrick Gunning (University of Toronto, Mississauga campus). BMIC line BT478 was treated with log dilutions of the inhibitors and IC_50_ values determined by assessing cell proliferation. PG-S3-002 was chosen for further assessment for inhibition of tumor formation. BT478 cells were dissociated to single cells and 1,000,000 cells replated in TSM. Cells were treated with either PG-S3-002 at IC_90_ or DMSO for 4 days, harvested, and injected intracranially into NOD/SCID mice (*n* = 3). Mice were sacrificed at 4 weeks and the brains harvested and embedded in paraffin for H&E staining, and tumor size determined from each section.

### Meta-analysis of gene-expression profiles in NSCLC

We analyzed 11 publicly available NSCLC gene expression datasets (Supplementary Table 3), originally from studies on tissue samples obtained from surgically resected human lung tumors and containing at least one sample of noncancerous normal tissue for comparison. To enable uniform processing and analysis of all the datasets and thus to improve comparability of results, we chose only datasets that were produced by using Affymetrix platforms. Each of the datasets was first separately normalized and summarized using Bioconductor project’s package gcrma (GeneChip Robust Multiarray Averaging) [77]. For the each individual dataset, we then evaluated differential expression of the genes using Bioconductor’s limma package [28]. Based on expression fold change, genes were classified as either up- or downregulated, and then ranked according to statistical significance, which was evaluated by *q*-value (adjusted *p*-value). Analyzing 11 datasets we thus obtained 22 different rankings, 11 rankings for upregulated genes and 11 for downregulated ones. To identify consistently deregulated genes obtained rankings were subjected to robust rank aggregation analysis implemented as an R package RobustRankAggreg [79]. This analysis detects genes which are ranked consistently better than expected under a null hypothesis of uncorrelated inputs and assigns a *p*-value as a significance score for each gene. The stability of resulting significance scores was then assessed by the leave-one-out validation, in which the same analysis was repeated 11 times, each time excluding one of the rankings. Acquired *p*-values from each round were finally averaged into corrected *p*-value. Genes whose significance score was greater than chosen threshold (*p* < 0.05) were further considered as consistently deregulated genes.

### Assembly of the STAT3- hsa-mir-21 regulatory network

Both STAT3 and hsa-mir-21 are very potent regulators of expression targeting wide range of genes. To identify their targets and relationships among them, we collated data from multiple independent sources. Knowledge of human transcription factors (TF) and their respective targets was obtained from four different databases, namely: ChEA (ChIP Enrichment Analysis) database [80], ITFP [81] (Integrated Transcription Factor Platform), PAZAR [82], TRED [83] (Transcriptional Regulatory Element Database). These data were either downloaded as a flat file (ITFP, PAZAR), manually collected (ITFP), or acquired from the web-based interactive application (ChEA). Additional data were obtained from lists of TF:target pairs from human fetal lung provided by Neph *et al*. [84]. Names of TFs and their respective targets as obtained from these databases were first standardized according to HGNC symbol checker [http://www.genenames.org/cgi-bin/symbol_checker] and then concatenated into the single list comprising all the unique TF:target pairs. We used mirDIP [85] (microRNA Data Integration Portal, version 2.0) to acquire a list of targets of hsa-mir-21. In our search we considered only miRNA–target relationships which fall among the top third of the most plausible predictions from at least three different databases. HGNC symbol checker then standardized names of the targeted genes. As a result we obtained a network comprising STAT3-hsa-mir-21 and their targets represented by nodes, and regulatory relationships between these as edges.

### *In silico* analysis of miR 21 in lung adenocarcinoma

#### Patients and samples

All TCGA data was obtained through the TCGA data portal (https://tcga-data.nci.nih.gov/tcga/) on September 15th 2014. Level 3 gene or miRNA expression data, as well as de-identified patient clinical data, was used for our analysis.

#### miR-21 co-expression data

We calculated Pearson distance between miR-21 and all the Genes/miRNAs to identify features that were inversely co-expressed with miRNA-21 in lung adenocarcinoma samples. The top 250 inversely co-expressed genes/miRNAs are included as Supplementary Table 4.

#### Survival analysis

Survival analysis was completed in R and survival curves were graphed using Graphpad Prism 5.

### Statistical analysis

Replicates from at least three samples were compiled for each experiment, unless otherwise specified in figure legends. Respective data represent mean ± SD with *n* values listed in figure legends. Student’s *t-test* analyses and 2-way *ANOVA* analysis were performed using GraphPad Prism 5. *P* < 0.05 was considered significant.

## SUPPLEMENTARY DATA


