# Supplementary material for: STAT3 pathway regulates lung-derived brain metastasis initiating cell capacity through miR-21 activation
Source: Oncotarget. 2015 Jul 25;6(29):27461–77. doi: 10.18632/oncotarget.4742 (PMC4695002; doi:10.18632/oncotarget.4742)
Supplement: Supplementary file 1 [file oncotarget-06-27461-s001.pdf]

SUPPLEMENTARY DATA

REFERENCES

1. Asangani IA, Rasheed SA, Nikolova DA, Leupold JH, Colburn NH, Post S et al. MicroRNA-21 (miR-21) post-transcriptionally downregulates tumor suppressor Pdcd4 and stimulates invasion, intravasation and metastasis in colorectal cancer. Oncogene 2008; 27:2128–2136.
2. de Oliveira PE, Zhang L, Wang Z, Lazo JS. Hypoxia-mediated regulation of Cdc25A phosphatase by p21 and miR-21. Cell cycle 2009; 8:3157–3164.
3. Gabriely G, Wurdinger T, Kesari S, Esau CC, Burchard J, Linsley PS et al. MicroRNA 21 promotes glioma invasion by targeting matrix metalloproteinase regulators. Molecular and cellular biology 2008; 28:5369–5380.
4. Gastwirt RF, Slavin DA, McAndrew CW, Donoghue DJ. Spy1 expression prevents normal cellular responses to DNA damage: inhibition of apoptosis and checkpoint activation. The Journal of biological chemistry 2006; 281: 5425–35435.
5. Liu M, Wu H, Liu T, Li Y, Wang F, Wan H et al. Regulation of the cell cycle gene, BTG2, by miR-21 in human laryngeal carcinoma. Cell research 2009; 19:828–837.
6. Liu M, Tang Q, Qiu M, Lang N, Li M, Zheng Y et al. miR-21 targets the tumor suppressor RhoB and regulates proliferation, invasion and apoptosis in colorectal cancer cells. FEBS letters 2011; 585:2998–3005.
7. Lu Z, Liu M, Stribinskis V, Klinge CM, Ramos KS, Colburn NH et al. MicroRNA-21 promotes cell transformation by targeting the programmed cell death 4 gene. Oncogene 2008; 27:4373–4379.
8. Luis-Ravelo D, Anton I, Zandueta C, Valencia K, Pajares MJ, Agorreta J et al. RHOB influences lung adenocarcinoma metastasis and resistance in a host-sensitive manner. Molecular oncology 2014; 8:196–206.
9. McAndrew CW, Gastwirt RF, Meyer AN, Porter LA, Donoghue DJ. Spy1 enhances phosphorylation and degradation of the cell cycle inhibitor p27. Cell cycle 2007; 6:1937–1945.
10. Meng F, Henson R, Wehbe-Janek H, Ghoshal K, Jacob ST, Patel T. MicroRNA-21 regulates expression of the PTEN tumor suppressor gene in human hepatocellular cancer. Gastroenterology 2007; 133:647–658.
11. Papagiannakopoulos T, Shapiro A, Kosik KS. MicroRNA-21 targets a network of key tumor-suppressive pathways in glioblastoma cells. Cancer research 2008; 68:8164–8172.
12. Sayed D, Rane S, Lypowy J, He M, Chen IY, Vashistha H et al. MicroRNA-21 targets Sprouty2 and promotes cellular outgrowths. Molecular biology of the cell 2008; 19:3272–3282.
13. Schramedei K, Morbt N, Pfeifer G, Lauter J, Rosolowski M, Tomm JM et al. MicroRNA-21 targets tumor suppressor genes ANP32A and SMARCA4. Oncogene 2011; 30: 2975–2985.
14. Watanabe T, Semba S, Yokozaki H. Regulation of PTEN expression by the SWI/SNF chromatin-remodelling protein BRG1 in human colorectal carcinoma cells. British journal of cancer 2011; 104:146–154.
15. Zhu S, Si ML, Wu H, Mo YY. MicroRNA-21 targets the tumor suppressor gene tropomyosin 1 (TPM1). The Journal of biological chemistry 2007; 282:14328–14336.

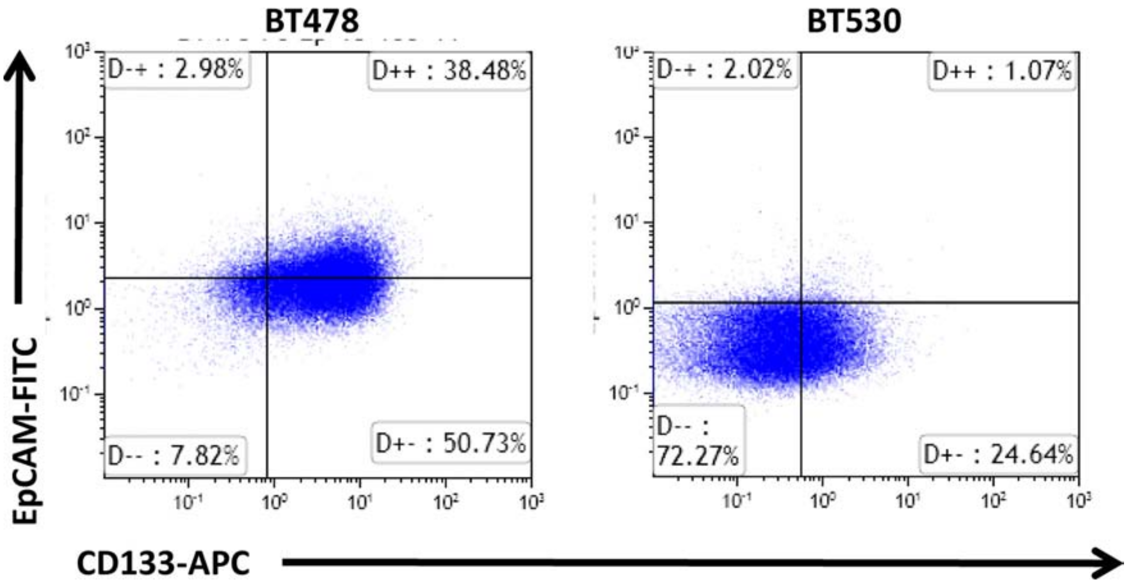

Supplementary Figure S1: CSC marker CD133 and epithelial marker EpCAM expression for BT478 and BT530 was assessed by flow cytometry.

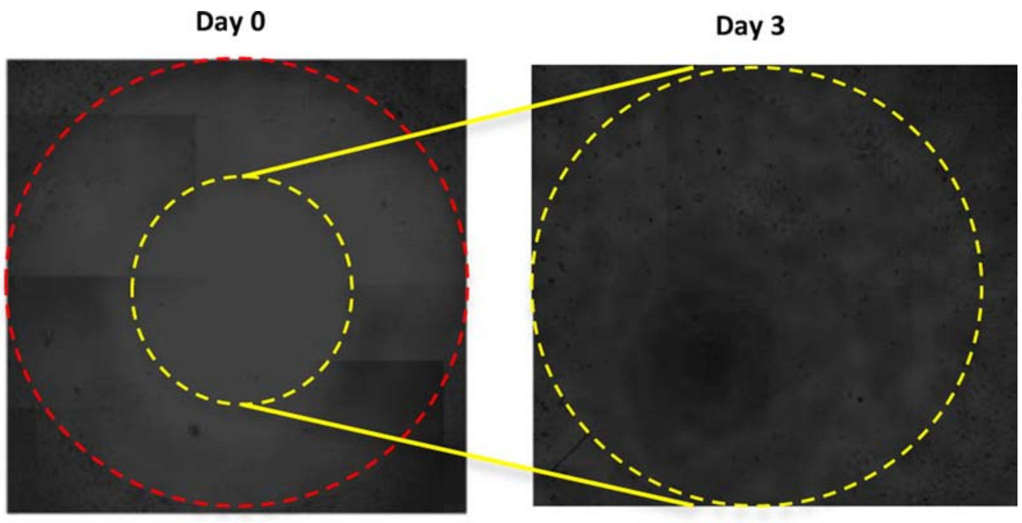

Supplementary Figure S2: Representative phase contrast images of zone exclusion assay. Red line is outline of cell border at day 0, yellow line is cell border at day 3.

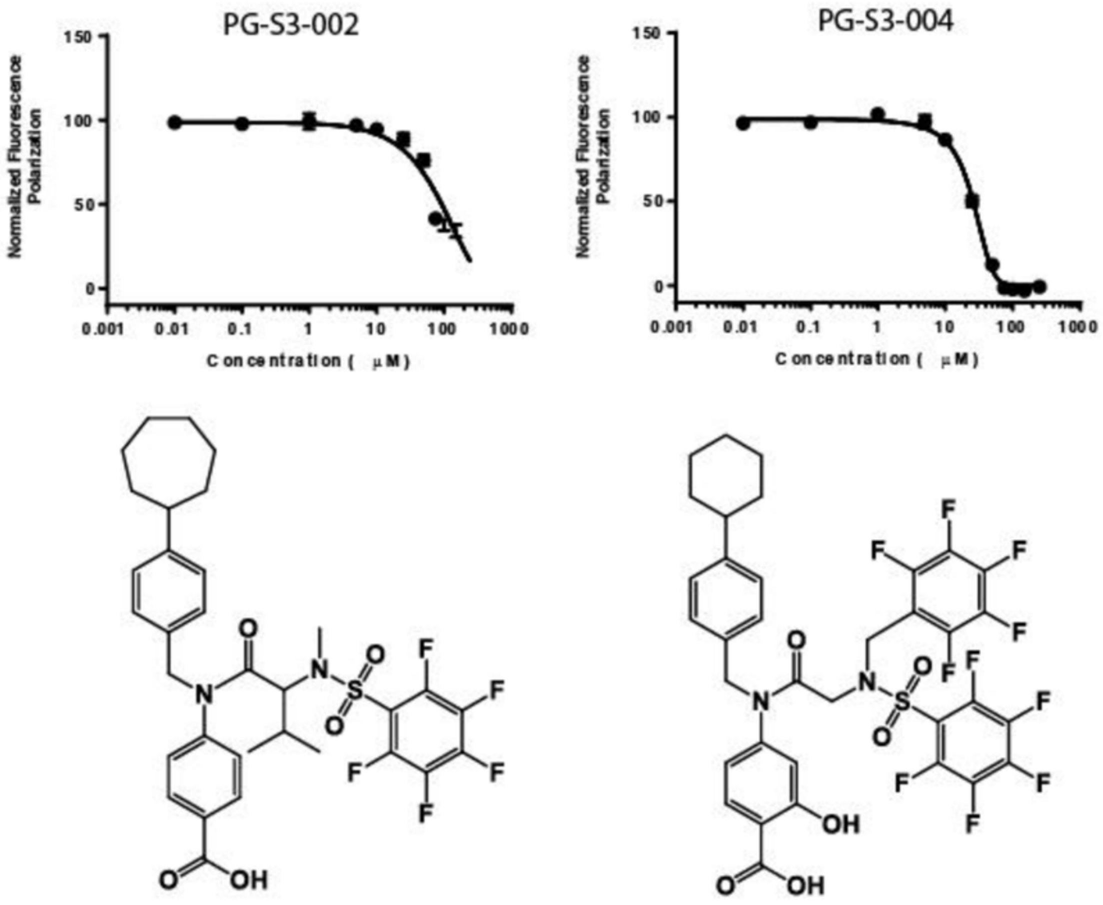

Compound ID	IC50 (µM)	95% Confidence Interval	Ki (µM)
PG-S3-002	88	75.9 to 101.7	45 ± 7
PG-S3-004	25	23.6 to 26.4	13 ± 0.7

Supplementary Figure S3: Determination of inhibitory constants for lead molecules using Fluorescence Polarization (FP) assay. Competitive binding FP assays were carried out for PG-S3-002 and PG-S3-004 (BP-5-087 FP previously determined by Gunning et al.)75,76 employing STAT3 and fluoresceinated-phosphopeptide 5-FAM-GpYLPQTV. Respective Ki's and IC50 values were derived from the appended competitive displacement curves and consolidated in the inset table. The assay results indicate that this family of molecules exhibits high affinity for the STAT3 protein.

Supplementary Table S1. List of antibodies and dyes used for flow cytometry and sorting

Antibody or Dye	Company	Amt/test (µL)	Isotype Control	Ex. (nm)	Em. (nm)
7-AAD Viability Dye	Beckman Coulter	10	N/A	546	647
Human CD133/2 APC	Miltenyi Biotec	10	IgG2b APC	650	660
EpCAM FITC	Miltenyi Biotec	10	IgG2a FITC	480	578
Mouse IgG2a FITC	R&D Systems	10	N/A	495	519
Mouse IgG2b APC	Miltenyi Biotec	10	N/A	650	660

Ex = excitation wavelength; Em = emission wavelength

Supplementary Table S2. List of qRT-PCR primers and their corresponding reverse and forward sequences

Gene	Forward Primer	Reverse Primer
GAPDH	TGCACCACCAACTGCTTAGC	GGCATGGACTGTGGTCATGAG
Stat3	AGGGTACATCATGGGCTTTATC	CTCCTTCTTTGCTGCTTTCAC
BTG2	CGTGAGCGAGCAGAGGCT TAAG	TGGACGGCTTTTCGGGAA
CDKN1A	GGCAGACCAGCATGACAGATT TC	CGGATTAGGGCTTCCTCTTGG
SMARCA4	GGGTAGCAGCAGATGTAGTTT C	CCAGTCACAAACAGTCCTACA G
ANP32A	TCCCTGTAAATGCGATAGCTA AG	GGGAAATACCAGGAAACGTAA GA
RECK	GCTCGGTTTGTTGCAGTTATG	ATCTGAGATGGACCAGGAGAA
PDCD4	GAGTACCAGTGTTGGCAGTATC	GTCCCACAAAGGTCAGAAAGA
RHOB	GGAGCTTGATATCCCTTGTCTG	CACCCATCACCACCCTTAAATA
TIMP3	TTTGCCCTTCTCCTCCAATAC	TCTTTCACACACCTTGAGTCT ATC
HNRPK	GGTGATCTTGGTGGACCTATTATT	TAATCCGCTGACCACCTTTG
SPRY2	TGTGGCAAGTGCAAATGTAAG	CAGCATACACAAGTCCCATAGT
SPRY1	GCCATCCACTTGAGGGTATT	GTAGTCTGGATGTGGGTGTATG

Supplementary Table S3. Gene expression datasets. Summary of gene expression datasets which were subjected to gene-expression meta-analysis

Platform	Cancer type	No. of samples	Author	Year	Ref. and notes
Hu6800	ADC	96/10	Beer et al.	2002	Beer, David G., et al. "Gene-expression profiles predict survival of patients with lung adenocarcinoma." Nature medicine 8.8 (2002):816–824.
HG-U95Av2	ADC	228/17	Bhattacharjee et al.	2001	Bhattacharjee, Arindam, et al. "Classification of human lung carcinomas by mRNA expression profiling reveals distinct adenocarcinoma subclasses." Proceedings of the National Academy of Sciences 98.24 (2001):13790–13795.
HG-U133p2	ADC, SCC, LCC	156/64	Hou et al.	2010	Hou, Jun, et al. "Gene expression-based classification of non-small cell lung carcinomas and survival prediction." PloS one 5.4 (2010):e10312.
HG-U133a	ADC	107/50	Landi et al.	2008	Landi, Maria Teresa, et al. "Gene expression signature of cigarette smoking and its role in lung adenocarcinoma development and survival." PloS one 3.2 (2008):e1651.
HG-U133p2	ADC	246/20	Okayama et al.	2012	Okayama, Hirokazu, et al. "Identification of genes upregulated in ALK-positive and EGFR/KRAS/ALK-negative lung adenocarcinomas." Cancer research 72.1 (2012):100–111.
HG-Focus	ADC, SCC	25/5	Rohrbeck et al.	2008	Rohrbeck, Astrid, et al. "Gene expression profiling for molecular distinction and characterization of laser captured primary lung cancers." J Transl Med 6 (2008):69.
HG-U133p2	ADC, SCC	91/45	Sanchezpalencia et al.	2011	Sanchez-Palencia, Abel, et al. "Gene expression profiling reveals novel biomarkers in nonsmall cell lung cancer." International Journal of Cancer 129.2 (2011):355–364.
HG-U95Av2	ADC	39/19	Stearman et al.	2005	Stearman, Robert S., et al. "Analysis of orthologous gene expression between human pulmonary adenocarcinoma and a carcinogen-induced murine model." The American journal of pathology 167.6 (2005):1763–1775.
HG-U133a	ADC	54/27	Su et al	2007	Su, Li-Jen, et al. "Selection of DDX5 as a novel internal control for Q-RT-PCR from microarray data using a block bootstrap re-sampling scheme." BMC genomics 8.1 (2007):140.
HG-U133a	ADC	50/20	Girard et al.	2011	Unpublished data, GEO a.n. - GSE31547
HG-U133a	ADC	58/9	Yap et al.	2005	Yu, Lei, et al. "Early detection of lung adenocarcinoma in sputum by a panel of microRNA markers." International Journal of Cancer 127.12 (2010):2870–2878.

Supplementary Table S4. Top 250 inversely co-expressed genes/miRNAs in lung adenocarcinoma samples

Inverse Correlation with miR-21	Correlation Co-efficient	*P*-value
FGR	−0.2137634	6.23E-06
C2orf39	−0.2137697	6.23E-06
CSRP2BP	−0.2137713	6.23E-06
LIPA	−0.2138031	6.21E-06
LEPR	−0.2138322	6.19E-06
RIMKLA	−0.2139388	6.12E-06
RP1-177G6.2	−0.2139501	6.11E-06
TGFBRAP1	−0.213967	6.1E-06
MEOX1	−0.2140475	6.05E-06
MAN1A1	−0.2140548	6.05E-06
ZDHHC3	−0.2140701	6.04E-06
TIMP3	−0.2140774	6.03E-06
USP8	−0.2141791	5.97E-06
GPBAR1	−0.2142519	5.93E-06
SYNE1	−0.2142598	5.92E-06
C6orf72	−0.2143394	5.87E-06
NIPSNAP3A	−0.2143462	5.87E-06
GIMAP1	−0.2144321	5.82E-06
DYX1C1	−0.2144417	5.81E-06
TYRP1	−0.2145129	5.77E-06
C4orf19	−0.2146031	5.72E-06
SPATA1	−0.2146151	5.71E-06
HELQ	−0.2146483	5.69E-06
ZNF620	−0.2146789	5.67E-06
TMEM107	−0.2147763	5.62E-06
DCTN1	−0.2148942	5.55E-06
DMD	−0.2149165	5.54E-06
GATA5	−0.214938	5.52E-06
CRTC3	−0.21499	5.49E-06
FAM183A	−0.2150234	5.48E-06
RAB4A	−0.2151681	5.4E-06
SNCAIP	−0.2152695	5.34E-06
DTHD1	−0.2152724	5.34E-06
C11orf66	−0.2153902	5.27E-06

(*Continued*)

Inverse Correlation with miR-21	Correlation Co-efficient	*P*-value
GIMAP4	−0.2153957	5.27E-06
RNF152	−0.2155458	5.19E-06
GORASP1	−0.2156193	5.15E-06
GP5	−0.2156493	5.13E-06
TSPYL4	−0.2156529	5.13E-06
IHH	−0.2157948	5.06E-06
CDS2	−0.2158304	5.04E-06
PCDHB1	−0.2158562	5.03E-06
HNRNPH2	−0.2159212	4.99E-06
GKAP1	−0.2159392	4.98E-06
ISOC1	−0.2159428	4.98E-06
AR	−0.2159433	4.98E-06
KNCN	−0.2159556	4.97E-06
SH3BGRL2	−0.2161892	4.86E-06
DNAH10	−0.2162708	4.82E-06
hsa-mir-29c	−0.2162953	4.8E-06
CRTAP	−0.2163165	4.79E-06
HSF2	−0.2163317	4.78E-06
CTAGE1	−0.2164685	4.72E-06
C6orf165	−0.2165042	4.7E-06
FLJ10357	−0.2165216	4.69E-06
WDR19	−0.2165717	4.67E-06
MYO1C	−0.2165918	4.66E-06
MIPEP	−0.2166741	4.62E-06
TPRG1L	−0.2166744	4.62E-06
hsa-mir-3619	−0.2166798	4.62E-06
RCBTB1	−0.2166806	4.62E-06
DIRC2	−0.2167577	4.58E-06
ITPR1	−0.2167633	4.58E-06
GAS7	−0.2169031	4.51E-06
FIG4	−0.2169034	4.51E-06
SENP8	−0.2169084	4.51E-06
EFCAB6	−0.2169473	4.49E-06
MAP9	−0.2170261	4.45E-06
IFT172	−0.2170481	4.44E-06
SEC23B	−0.2170689	4.43E-06

(*Continued*)

Inverse Correlation with miR-21	Correlation Co-efficient	*P*-value
FAM134B	−0.2171249	4.41E-06
LIN52	−0.2171234	4.41E-06
CNR1	−0.217155	4.39E-06
SIN3A	−0.2172971	4.33E-06
ACADM	−0.2173316	4.31E-06
TMSB15A	−0.217397	4.28E-06
hsa-mir-501	−0.2174483	4.26E-06
RNF185	−0.2175804	4.2E-06
FBLN1	−0.2176092	4.19E-06
SETX	−0.2176477	4.17E-06
PRRG3	−0.2176794	4.16E-06
FAM105A	−0.217759	4.13E-06
RNF122	−0.2177864	4.11E-06
GPRASP1	−0.2178293	4.1E-06
C1orf150	−0.2178439	4.09E-06
FAM188A	−0.2178562	4.08E-06
CCL24	−0.2179691	4.04E-06
ACR	−0.2180029	4.02E-06
CRMP1	−0.2180225	4.01E-06
OSR1	−0.2180289	4.01E-06
SHH	−0.218051	4E-06
CCDC65	−0.2181219	3.97E-06
ZFP1	−0.2181635	3.96E-06
AXIN2	−0.2182355	3.93E-06
STAG1	−0.2182684	3.91E-06
CACHD1	−0.2182727	3.91E-06
ZNF192	−0.218289	3.9E-06
SPRY2	−0.2183687	3.87E-06
MTMR9	−0.2184087	3.86E-06
CHST10	−0.2184182	3.85E-06
LPPR5	−0.218443	3.84E-06
CDHR3	−0.2184675	3.83E-06
MAP1LC3B2	−0.218763	3.72E-06
WBP4	−0.2187828	3.71E-06
C9orf171	−0.2188375	3.69E-06
ID3	−0.2188646	3.68E-06

(*Continued*)

Inverse Correlation with miR-21	Correlation Co-efficient	*P*-value
hsa-mir-3940	−0.2189321	3.65E-06
ALDH2	−0.2189918	3.63E-06
CYB5A	−0.2190779	3.6E-06
AADAC	−0.2190835	3.59E-06
EFCAB1	−0.2190903	3.59E-06
DTWD2	−0.2192643	3.53E-06
C11orf58	−0.2193356	3.5E-06
NFYB	−0.2193862	3.48E-06
SAMHD1	−0.2194152	3.47E-06
CD68	−0.2194246	3.47E-06
PRMT8	−0.2194607	3.45E-06
CHST9	−0.2194863	3.44E-06
KIF26A	−0.2194889	3.44E-06
NMBR	−0.2195201	3.43E-06
FBXL7	−0.2195265	3.43E-06
STK33	−0.2195314	3.43E-06
C9orf153	−0.219538	3.43E-06
FRMPD1	−0.2195508	3.42E-06
FAM110B	−0.2195653	3.42E-06
ZMYND10	−0.2195903	3.41E-06
ZNF441	−0.2196303	3.39E-06
hsa-mir-92a-1	−0.2196761	3.38E-06
DYNC1LI1	−0.2196993	3.37E-06
DNAH9	−0.2197759	3.34E-06
TPT1	−0.2198786	3.31E-06
PLEKHA2	−0.2198789	3.31E-06
SLC4A1	−0.2198968	3.3E-06
GPD1L	−0.2199198	3.29E-06
C1orf88	−0.2200335	3.25E-06
ZNF20	−0.2200835	3.23E-06
FBXW2	−0.2201312	3.22E-06
SLC5A9	−0.2201332	3.22E-06
ATP8A2	−0.2201434	3.21E-06
KIF19	−0.2201704	3.21E-06
WDR16	−0.2202321	3.18E-06
TUBB1	−0.2202427	3.18E-06

(*Continued*)

Inverse Correlation with miR-21	Correlation Co-efficient	*P*-value
ZNF295	−0.2202431	3.18E-06
FRAT1	−0.220355	3.14E-06
KIAA0513	−0.2203605	3.14E-06
IRX1	−0.2204367	3.12E-06
ARHGAP28	−0.2204605	3.11E-06
KIDINS220	−0.2204624	3.11E-06
SPAG6	−0.2205465	3.08E-06
hsa-mir-607	−0.2205482	3.08E-06
SERGEF	−0.2207014	3.03E-06
SLC25A38	−0.220726	3.02E-06
RAB36	−0.2209666	2.95E-06
C1orf129	−0.2210794	2.91E-06
MBNL3	−0.2211417	2.89E-06
TOP2B	−0.2212776	2.85E-06
B3GALNT1	−0.2213013	2.84E-06
EIF3F	−0.2213536	2.83E-06
NUCB1	−0.2213637	2.83E-06
LOC144571	−0.2213992	2.82E-06
ACP5	−0.2214151	2.81E-06
KCNJ11	−0.2214198	2.81E-06
ROCK1	−0.2214327	2.81E-06
ENG	−0.2214741	2.79E-06
ALOX15	−0.2214765	2.79E-06
RAB39B	−0.2214969	2.79E-06
SEC14L4	−0.2217393	2.72E-06
GRINL1A	−0.2218065	2.7E-06
RNF6	−0.2218022	2.7E-06
KCNRG	−0.2218342	2.69E-06
GALC	−0.2218923	2.67E-06
SECISBP2L	−0.2219035	2.67E-06
CYP4V2	−0.2219426	2.66E-06
C8orf84	−0.2219631	2.65E-06
PCDH20	−0.2219737	2.65E-06
OSCAR	−0.2220473	2.63E-06
KBTBD11	−0.2220564	2.63E-06
TTC19	−0.2220586	2.63E-06

(*Continued*)

Inverse Correlation with miR-21	Correlation Co-efficient	*P*-value
CDH23	−0.2222177	2.58E-06
FHDC1	−0.222297	2.56E-06
B9D2	−0.2223257	2.55E-06
C17orf108	−0.2223365	2.55E-06
CTR9	−0.2223921	2.53E-06
MECOM	−0.2224532	2.52E-06
ZBTB44	−0.2227983	2.43E-06
C10orf105	−0.22289	2.4E-06
RGS22	−0.2229588	2.39E-06
ZBTB3	−0.2229643	2.38E-06
hsa-mir-769	−0.223015	2.37E-06
KLF2	−0.223015	2.37E-06
RASSF5	−0.2230249	2.37E-06
hsa-mir-320c-1	−0.2230523	2.36E-06
ZBTB7C	−0.2232174	2.32E-06
KIAA1958	−0.2232485	2.31E-06
PPPDE1	−0.2233126	2.3E-06
SCD5	−0.2233911	2.28E-06
ARHGEF2	−0.2234129	2.27E-06
C4orf3	−0.2235013	2.25E-06
MT1M	−0.2235185	2.25E-06
ICAM2	−0.2235228	2.25E-06
ANKRD1	−0.2237605	2.19E-06
DENND5A	−0.2238421	2.17E-06
CLEC4M	−0.2238968	2.16E-06
SYNPO2L	−0.2239025	2.16E-06
TNFSF12	−0.2239337	2.15E-06
SEMA6D	−0.2241106	2.11E-06
MITF	−0.224142	2.1E-06
FAM124A	−0.2242278	2.08E-06
C2orf67	−0.2242396	2.08E-06
KIAA0564	−0.2242519	2.08E-06
JDP2	−0.2244195	2.04E-06
GJA4	−0.2244906	2.02E-06
CCDC152	−0.2245314	2.02E-06
SLC24A3	−0.2247037	1.98E-06

(*Continued*)

Inverse Correlation with miR-21	Correlation Co-efficient	*P*-value
C11orf88	−0.2247349	1.97E-06
PHF17	−0.2247447	1.97E-06
FXR2	−0.2249092	1.94E-06
LOC285796	−0.2249177	1.93E-06
SLC35A1	−0.2249266	1.93E-06
FAM188B	−0.2249584	1.93E-06
ST7L	−0.2250667	1.9E-06
TMEM125	−0.2251253	1.89E-06
LRRC48	−0.2251545	1.88E-06
BIVM	−0.2252641	1.86E-06
CYGB	−0.2252953	1.86E-06
CAMP	−0.2252963	1.86E-06
PPP2R5A	−0.2253875	1.84E-06
PCDHGA9	−0.2255059	1.81E-06
C6orf97	−0.2255288	1.81E-06
USP54	−0.2255463	1.81E-06
hsa-mir-92b	−0.2255781	1.8E-06
SLC22A3	−0.225594	1.8E-06
FAM82A1	−0.2255948	1.8E-06
PPP1R14A	−0.225642	1.79E-06
UACA	−0.2257861	1.76E-06
RNF11	−0.2258238	1.75E-06
LCORL	−0.2259605	1.73E-06
KIF3B	−0.2259775	1.72E-06
SWAP70	−0.2260871	1.7E-06
ATF7	−0.2261481	1.69E-06
TNFRSF19	−0.2261629	1.69E-06
GSTA4	−0.2261774	1.69E-06
FAM43A	−0.2262422	1.68E-06
hsa-mir-200c	−0.2262697	1.67E-06
KIAA0427	−0.2263043	1.66E-06
C1orf194	−0.2263314	1.66E-06
C6orf225	−0.2264753	1.63E-06
NHLRC4	−0.2266156	1.61E-06
UNC13B	−0.2267202	1.59E-06
DLL1	−0.2267784	1.58E-06

(*Continued*)

Supplementary Table S5. mir-21 upregulation across various miRNA profiling studies

Fold Change	*P*-value	Histology	No. of samples (total/normal)	Author	Year	Ref.
2.9	<0.001	SCC, ADC	52/24	Boeri et al.	2011	Boeri, Mattia, et al. "MicroRNA signatures in tissues and plasma predict development and prognosis of computed tomography detected lung cancer." Proceedings of the National Academy of Sciences 108.9 (2011):3713–3718.
4.7	1.08E-005	ADC	20/10	Cho et al.	2009	Cho, William, Andrew SC Chow, and Joseph SK Au. "Restoration of tumour suppressor hsa-miR-145 inhibits cancer cell growth in lung adenocarcinoma patients with epidermal growth factor receptor mutation." European Journal of Cancer 45.12 (2009):2197–2206.
3.5	6.13E-005	ADC	20/8	Crawford et al.	2009	Crawford, Melissa, et al. "MicroRNA 133B targets pro-survival molecules MCL-1 and BCL2L2 in lung cancer." Biochemical and biophysical research communications 388.3 (2009):483–489.
3.2	2.00E-004	SCC, ADC	12/6	Gao et al.	2010	Gao, Wen, et al. "Deregulated expression of miR-21, miR-143 and miR-181a in non small cell lung cancer is related to clinicopathologic characteristics or patient prognosis." Biomedicine & Pharmacotherapy 64.6 (2010):399–408.
2.4	2.30E-009	ADC	112/56	Jang et al.	2012	Jang, Jin Sung, et al. "Increased miR-708 expression in NSCLC and its association with poor survival in lung adenocarcinoma from never smokers." Clinical Cancer Research 18.13 (2012):3658–3667.
–	< 0.01	SCC, ADC	40/20	Puissegur et al.	2011	Puissegur, M. P., et al. "miR-210 is overexpressed in late stages of lung cancer and mediates mitochondrial alterations associated with modulation of HIF-1 activity." Cell Death & Differentiation 18.3 (2010):465–478.
2.4	3.00E-004	SCC, ADC	56/28	Seike et al.	2009	Seike, Masahiro, et al. "MiR-21 is an EGFR-regulated anti-apoptotic factor in lung cancer in never-smokers." Proceedings of the National Academy of Sciences 106.29 (2009):12085–12090.
2.3	4.11E-003	SCC, ADC	65/27	Vosa et al.	2011	Võsa, Urmo, et al. "Identification of miR-374a as a prognostic marker for survival in patients with early-stage nonsmall cell lung cancer." Genes, Chromosomes and Cancer 50.10 (2011):812–822.

Expression of mir-21 in tumours has been found significantly upregulated compared to normal tissue by 12 miRNA profiling studies. Table summarizes reported fold-change (if available) of mir-21 expression, together with its level of significance, as well as histology and number of samples that have been used.

(*Continued*)

Fold Change	*P*-value	Histology	No. of samples (total/normal)	Author	Year	Ref.
2.6	< 0.01	SCC, ADC	46/23	Wang et al.	2011	Wang, R., et al. “MicroRNA-451 functions as a tumor suppressor in human non-small cell lung cancer by targeting ras-related protein 14 (RAB14).” Oncogene 30.23 (2011):2644–2658.
1.7	< 0.01	SCC, ADC	30/15	Xing et al.	2010	Xing, Lingxiao, et al. “Early detection of squamous cell lung cancer in sputum by a panel of microRNA markers.” Modern Pathology 23.8 (2010):1157–1164.
–	1.00E-007	SCC, ADC	208/104	Yanaihara et al.	2006	Yanaihara, Nozomu, et al. “Unique microRNA molecular profiles in lung cancer diagnosis and prognosis.” Cancer cell 9.3 (2006):189–198.
2.6	< 0.01	ADC	40/20	Yu et al.	2010	Yu, Lei, et al. “Early detection of lung adenocarcinoma in sputum by a panel of microRNA markers.” International Journal of Cancer 127.12 (2010):2870–2878.

Supplementary Table S6. List of genes targeted by miR-21

Genes	Functional Role
TPM1	Tumor suppressor gene[15]
PDCD4	Reduce invasion and metastasis[1,7]
PTEN	Reduces Growth and Invasion[10]
RHOB	Tumor suppressor gene[6,8]
TIMP-3	Matrix metalloproteinase inhibitor[3]
RECK	Matrix metalloproteinase inhibitor[3]
HNRPK	Player in p53/TP53 response[11]
SPRY1	Inhibit DNA damage response pathways[4,9]
SPRY2	Inhibits cell migration[12]
CDKN1A	Cell cycle regulator[2]
ANP32A	Modulator of Apoptosis[13]
SMARCA4	Tumor suppressor[13,14]
BTG2	Anti-proliferative[5]
